# Diffuse-Type Tenosynovial Giant Cell Tumor of the Flexor Hallucis Longus Tendon Sheath: A Case Report

**DOI:** 10.7759/cureus.118245

**Published:** 2026-10-20

**Authors:** Anas Chbabou, Ayoub Mokhtari, Thomas Saliba

**Affiliations:** 1 Radiology, Erasmus Hospital, Brussels, BEL; 2 Radiology, H.U.B. (Hôpital Universitaire de Bruxelles), Brussels, BEL; 3 Radiology, Université Libre de Bruxelles, Brussels, BEL

**Keywords:** hemosiderin deposition, magnetic resonance imaging, musculo-skeletal pain, tenosynovial giant cell tumor, villonodular tumor of tendon sheat

## Abstract

The synovium can be the primary site for the development of various benign or malignant tumors. Tenosynovial giant cell tumor (TGCT), also known as giant cell tumor of the tendon sheath, is a benign but locally aggressive synovial neoplasm occurring in localized and diffuse forms. The localized form predominantly affects the hand, whereas the diffuse form mainly involves large joints; involvement of the flexor hallucis longus tendon sheath is rare. Magnetic resonance imaging (MRI) is the principal modality for demonstrating the hemosiderin deposits characteristic of this lesion, although histopathology remains necessary for definitive diagnosis. Surgical resection remains the standard treatment. We report the case of a 24-year-old man with 10 months of right ankle pain, related to a diffuse-type TGCT involving the flexor hallucis longus tendon sheath and adjacent ankle joints. Radiographs initially showed soft-tissue filling of Kager's triangle. Computed tomography arthrography demonstrated synovial thickening but was nonspecific; MRI then revealed the characteristic findings, and histopathology confirmed the diagnosis. This report aims to raise awareness of this rare location and its associated diagnostic pitfalls.

## Introduction

Tenosynovial giant cell tumor (TGCT), also known as giant cell tumor of the tendon sheath, is a benign yet locally aggressive neoplasm arising from synovial structures, including tendon sheaths, bursae, and joint synovium [1]. Population-based data from Myers and Masi indicate that the localized form is roughly five times more common than the diffuse form, with respective incidence rates near nine and under two new cases per million inhabitants each year [2]. It is characterized by recurrent genomic abnormalities involving the colony-stimulating factor 1 (*CSF1*) gene, driving macrophage recruitment within the tumor [3].

TGCT is classified into two forms: localized, the most common, presenting as a well-circumscribed nodular mass typically confined to a single tendon sheath or synovial compartment, and diffuse, characterized by an infiltrative, poorly circumscribed growth pattern that may extend across multiple synovial compartments, more aggressive and associated with a higher recurrence risk. As both forms share similar histological features, this distinction relies mainly on the growth pattern assessed on imaging and at surgery [1,4]. The localized form predominantly affects the hand and fingers, whereas the diffuse form mainly involves large joints, particularly the knee and hip [2]; involvement of the foot and ankle is less common [5]. Although TGCT can occur at any age, it most commonly presents between 30 and 50 years of age, with a two-to-one female predominance. While antecedent trauma has been proposed as a potential contributing factor, only approximately 15% of cases have a documented history of trauma [1].

Histologically, TGCT is composed of a proliferation of mononuclear stromal cells interspersed with multinucleated giant cells, with characteristic hemosiderin deposits [1,3]. Magnetic resonance imaging (MRI) is the principal imaging modality for demonstrating these deposits, although histopathological confirmation remains essential given the often nonspecific clinical presentation. The standard treatment is complete surgical excision, as local recurrence risk (nine to 45% across series) is primarily linked to incomplete excision and is higher in the diffuse form [6].

We report a case of diffuse-type TGCT involving the flexor hallucis longus tendon sheath and extending into the talocrural and subtalar joints, in which the post-traumatic context and a coexisting osteochondral lesion initially misled the diagnostic work-up.

## Case presentation

A 24-year-old man with no significant medical or surgical history was referred for right ankle pain that began immediately after a fall from a horse approximately 10 months earlier. The pain was mechanical, non-nocturnal, involved both the anterior and posterior aspects of the ankle, and was associated with a sensation of pressure and discomfort. It was aggravated by walking and running. The pain persisted despite conservative management with analgesics and physiotherapy. On clinical examination, the patient presented with mild, progressively increasing ankle swelling with edema, without restriction of ankle mobility or other signs of inflammation. Hallux flexion was painless, with preserved strength. Neurological and sensory examination was normal. Laboratory workup was unremarkable. 

Initial radiographs, performed at another institution one month before referral, had already revealed a soft-tissue density filling Kager's triangle associated with a heterogeneous density within the superomedial talar dome, raising suspicion for an osteochondral lesion of the talar dome associated with a joint effusion within the posterior recess of the talocrural joint (Figure 1).

**Figure 1:**
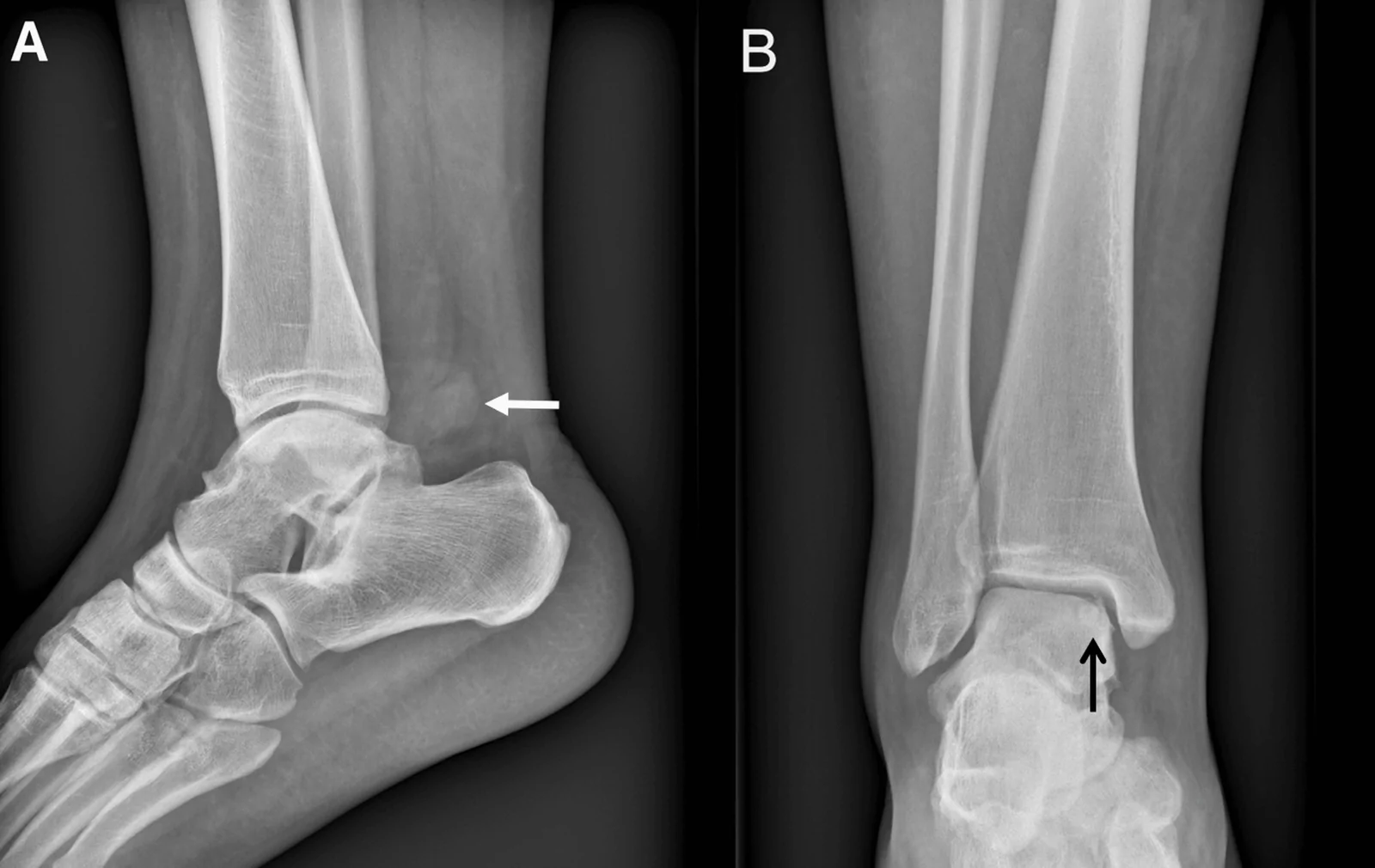
Initial right ankle radiographs (A) Initial lateral radiograph of the right ankle showing a soft-tissue density within Kager's triangle (white arrow). (B) Initial anteroposterior radiograph of the right ankle showing a heterogeneous density within the superomedial talar dome (black arrow), suggestive of an osteochondral lesion of the talar dome.

Pre-arthrography radiographs performed at our institution demonstrated an apparent increase in this soft-tissue density filling Kager's triangle compared to the initial radiographs; although the two lateral projections were not strictly identical, this increase appeared to reflect true progression (Figure 2). This finding was considered consistent with a joint effusion associated with an osteochondral lesion of the talar dome or bursitis. The hypothesis of a neoplastic process was not entertained initially. A post-traumatic hematoma was considered unlikely, as a hematoma would not be expected to enlarge over a one-month interval occurring ten months after the injury.

**Figure 2:**
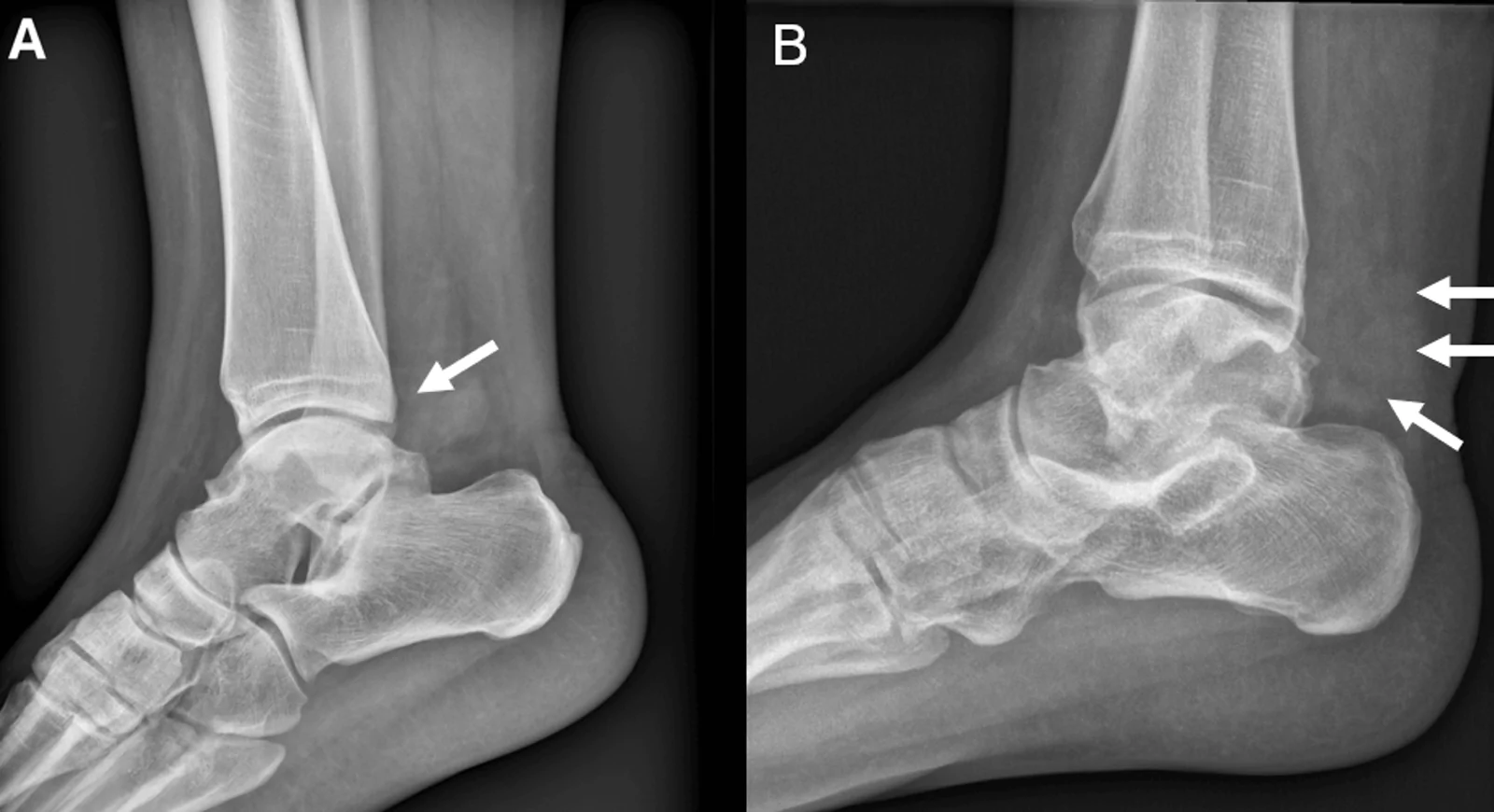
Initial and follow-up lateral ankle radiographs (A) Initial lateral radiograph of the right ankle, performed at another institution, showing a soft-tissue density filling Kager's triangle (arrow). (B) Pre-arthrography lateral radiograph of the ankle and foot, performed one month later at our institution, demonstrating an apparent increase in the extent of this soft-tissue density along the posterior ankle recess and adjacent to the calcaneus (arrows), which was initially suspected to represent a joint effusion associated with the osteochondral lesion of the talar dome.

A computed tomography (CT) arthrogram was performed before MRI, as an osteochondral lesion of the talar dome with associated joint effusion was the main suspected diagnosis at this stage. Iodinated contrast medium was injected into the talocrural joint, resulting in opacification of the talocrural joint as well as the subtalar joint and the flexor hallucis longus tendon sheath, indicating communication between these synovial compartments. The examination confirmed the osteochondral lesion at the superomedial talar dome, associated with an osteochondral fragment and cortical flattening, and also revealed irregular, multilobulated synovial thickening involving the flexor hallucis longus tendon sheath, extending into the talocrural and subtalar joints. This multicompartmental involvement was suggestive of an infiltrative rather than circumscribed synovial process. However, the CT arthrogram remained nonspecific regarding the tissue nature of the observed synovial thickening (Figure 3). Given the trauma mechanism and the onset of pain immediately after the fall, the osteochondral lesion was considered post-traumatic and unrelated to the synovial process.

**Figure 3:**
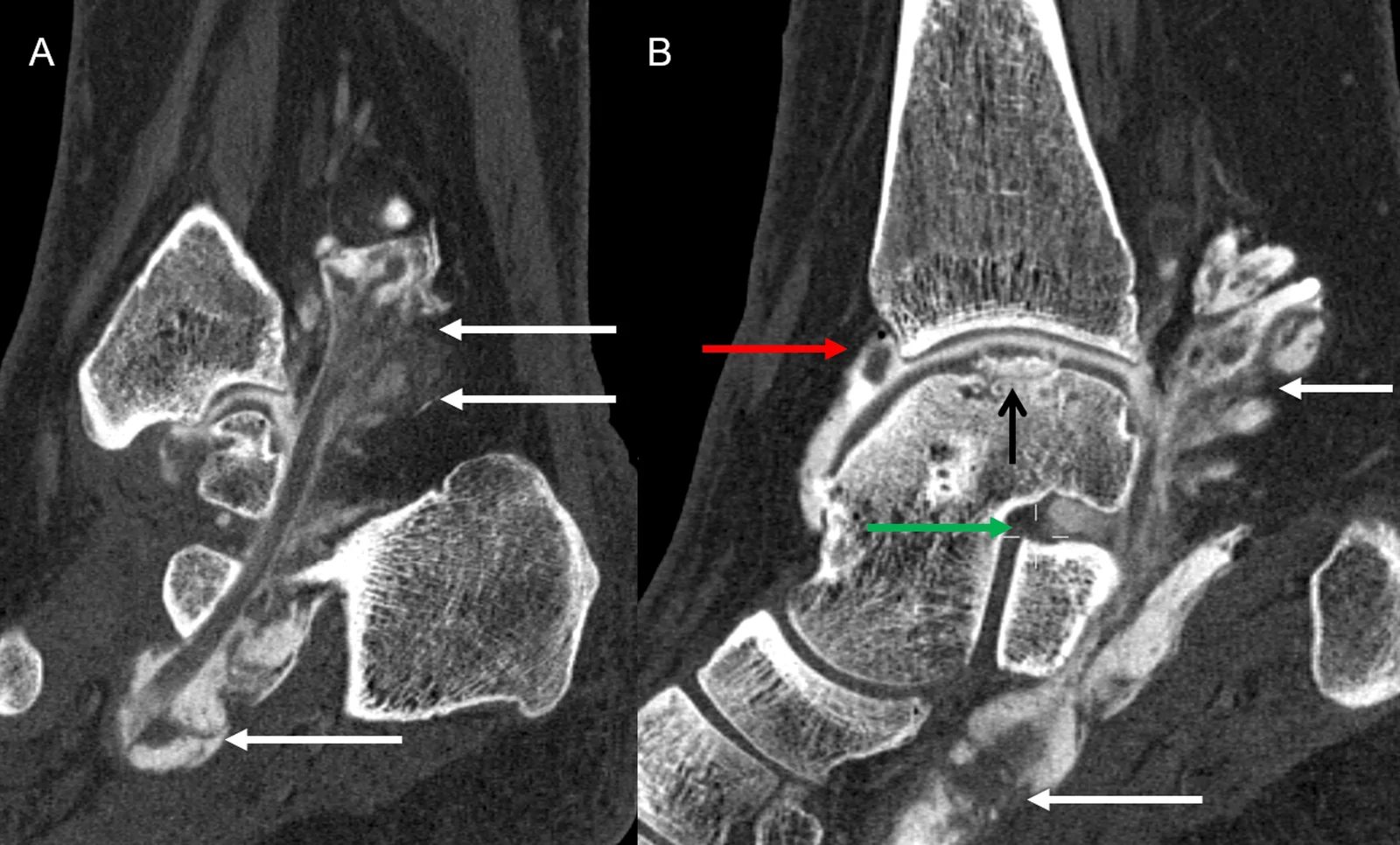
CT arthrogram of the right ankle Sagittal CT arthrogram images of the right ankle showing irregular, multilobulated synovial thickening of the flexor hallucis longus tendon sheath with associated peritendinous effusion (A, B, white arrows), extending into the talocrural joint (B, red arrow) and the subtalar joint (B, green arrow). An osteochondral lesion at the superomedial talar dome, associated with an osteochondral fragment and cortical flattening, is also seen (B, black arrow).

Given these unexpected findings and their nonspecific appearance, MRI was performed three weeks after the CT arthrogram. This revealed multilobulated thickening of the flexor hallucis longus tendon sheath, extending into the synovium of the talocrural and subtalar joints, hypointense on both T1- and T2-weighted sequences, with moderate enhancement following contrast administration (Figures 4, 5).

**Figure 4:**
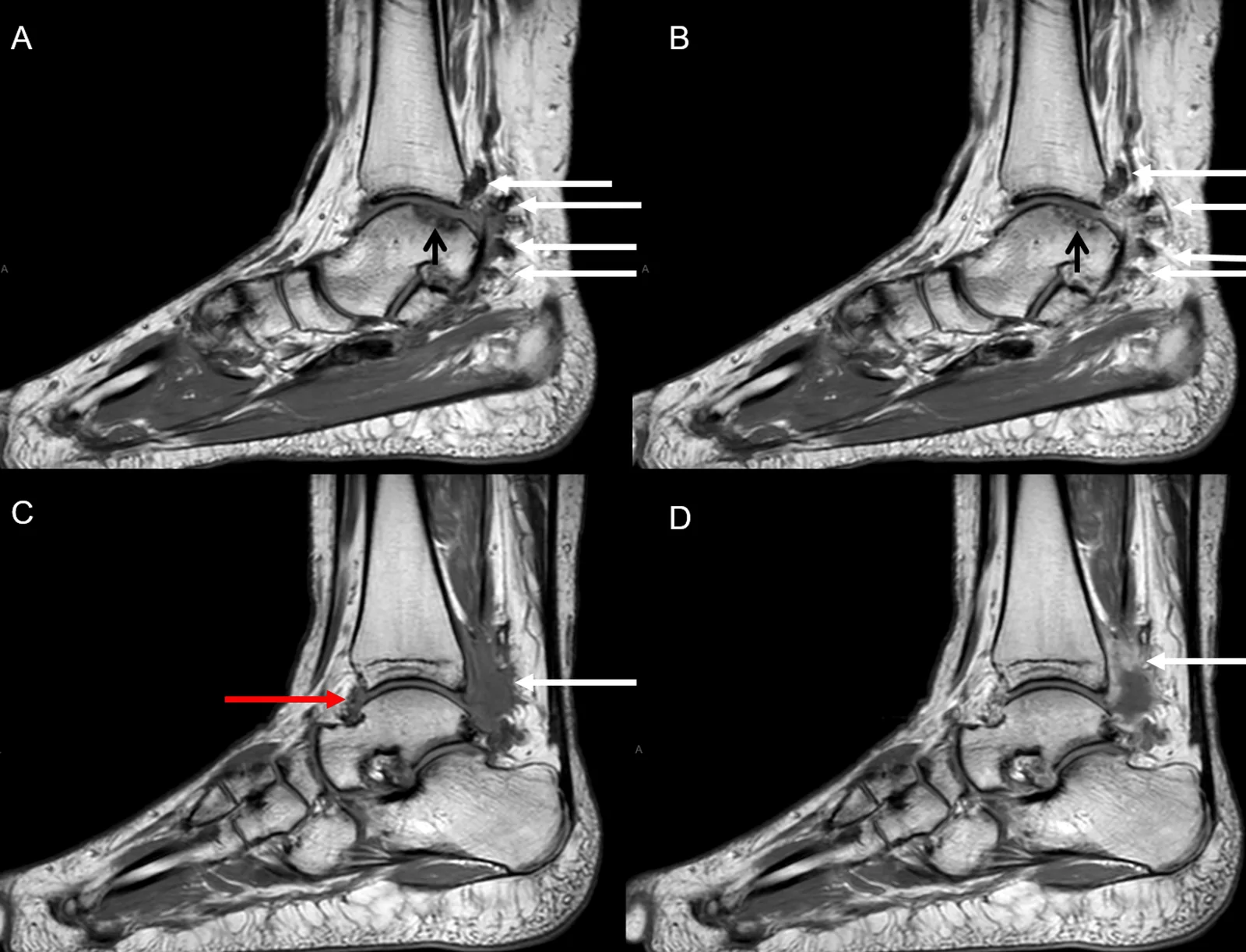
Sagittal T1-weighted MRI of the right ankle before and after contrast administration Sagittal T1-weighted images of the right ankle at two adjacent levels, obtained before (A, C) and after (B, D) intravenous gadolinium administration. A multilobulated thickening of the flexor hallucis longus tendon sheath (white arrows) is seen, extending into the synovium of the talocrural joint (C, red arrow), hypointense on precontrast images (A, C) and showing moderate enhancement following contrast administration (B, D). The osteochondral lesion of the talar dome is also seen (black arrows).

**Figure 5:**
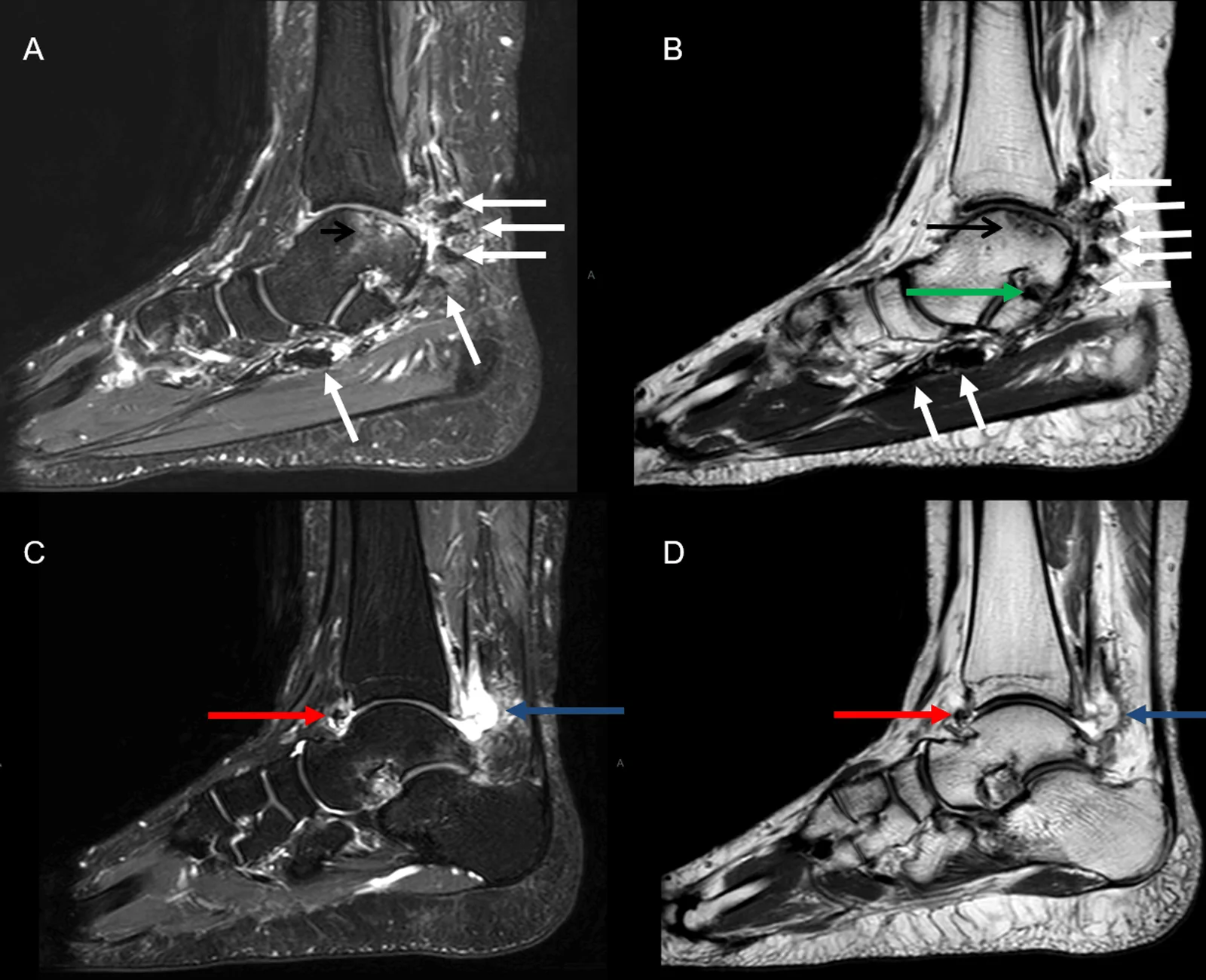
Sagittal T2 Dixon MRI of the right ankle at two adjacent levels Sagittal T2 Dixon water-only images (A, C) and corresponding in-phase images (B, D) of the right ankle at two adjacent levels. A multilobulated thickening of the flexor hallucis longus tendon sheath (A, B, white arrows) is seen, extending into the synovium of the talocrural joint (C, D, red arrows) and the subtalar joint (B, green arrow), demonstrating hypointense signal on both water-only and in-phase sequences. An associated joint effusion within the posterior recess of the talocrural joint (C, D, blue arrows) and the osteochondral lesion of the talar dome (A, B, black arrows) are also seen.

Gradient-echo sequences demonstrated magnetic susceptibility artifacts consistent with hemosiderin deposits secondary to repeated micro-hemorrhages, a finding considered highly suggestive of TGCT (Figure 6).

**Figure 6:**
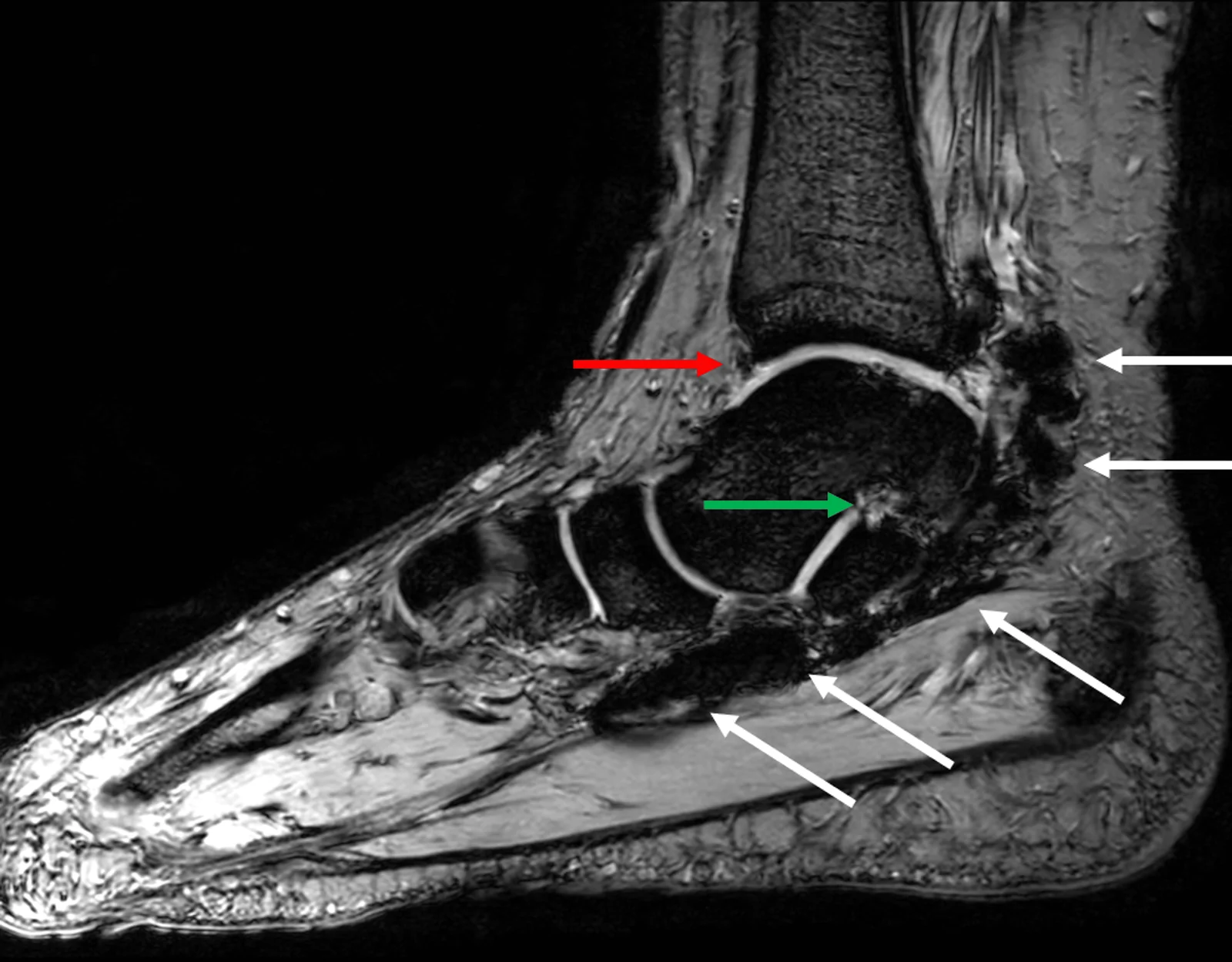
Sagittal gradient-echo MRI of the right ankle Sagittal gradient-echo image of the right ankle demonstrating pronounced magnetic susceptibility artifact (blooming) within the thickened flexor hallucis longus tendon sheath (white arrows), extending into the thickened synovium of the talocrural joint (red arrow) and the subtalar joint (green arrow), consistent with hemosiderin deposition secondary to repeated micro-hemorrhages.

Following multidisciplinary team discussion, and given the highly suggestive MRI findings, the orthopedic surgeon decided to proceed directly to excision of the tendon sheath tumor without preoperative biopsy. Treatment of the tumor was prioritized, while symptoms potentially related to the osteochondral lesion would be managed medically, with surgical treatment of the osteochondral lesion considered at a later stage if necessary. The patient was referred to the oncologic surgery department. Surgical excision was performed two weeks after the MRI, under regional nerve block anesthesia with a thigh pneumatic tourniquet, through combined anterior and posteromedial approaches to the ankle, using optical magnification. Intraoperatively, the lesion appeared as a brownish, multilobulated synovial mass measuring approximately 15 × 1 × 2 cm, poorly circumscribed and closely adherent to the flexor hallucis longus tendon sheath, from which it extended into the synovium of the talocrural and subtalar joints. The posterior tibial neurovascular bundle, which lies adjacent to the flexor hallucis longus tendon, was identified and protected throughout the dissection. The flexor hallucis longus tendon was preserved, synovectomy of the involved joint compartments was performed, and complete macroscopic excision was achieved. The wound was closed in layers.

Histological examination showed numerous papillary projections diffusely infiltrated by foamy histiocytes, multinucleated giant cells, and hemosiderin-laden macrophages, the latter correlating with the susceptibility artifacts observed on gradient-echo MRI. These findings confirmed the diagnosis of diffuse-type TGCT, and surgical margins were negative.

At two months postoperatively, the patient reported a reduction in ankle pain and discomfort and is currently undergoing physiotherapy to preserve joint and tendon mobility. A follow-up MRI has been scheduled at six months postoperatively to assess for recurrence.

## Discussion

TGCT is a rare, benign but locally aggressive synovial neoplasm. While the localized form most commonly involves the hand and fingers and the diffuse form large joints [2], involvement of the flexor hallucis longus tendon sheath is uncommon. This case illustrates the typical diagnostic pathway of TGCT in such a rare location: the nonspecific clinical presentation, with mechanical pain persisting for 10 months despite conservative management, mild swelling, and no inflammatory signs or restriction of mobility, often delays recognition of this condition, as also highlighted by Celayir et al. in their case of infrapatellar involvement, where initial clinical suspicion likewise did not favor TGCT [1]. In our patient, the post-traumatic context and the coexistence of an osteochondral lesion of the talar dome further misled the initial work-up, the soft-tissue density in Kager's triangle being attributed to a joint effusion rather than a mass. This illustrates a diagnostic pitfall: in a patient with persistent posterior ankle pain without inflammatory signs, a progressively increasing soft-tissue abnormality on radiographs may warrant MRI to exclude a synovial proliferative process, even when a traumatic explanation seems plausible. This lack of clinical specificity explains why diagnosis relies heavily on imaging, particularly MRI.

Notably, despite involvement of the flexor hallucis longus tendon sheath, hallux flexion was painless with preserved strength in our patient, so that tendon function did not point toward the lesion's origin, whereas the anterior and posterior ankle pain likely reflected its extension into the joint compartments. While the localized form predominantly affects the hand and fingers and the diffuse form affects large joints, cases reported in atypical locations, such as the infrapatellar region or the cervical spine, underscore the importance of maintaining a high index of suspicion for any atypical synovial mass, regardless of anatomical location [1,4].

In our case, as in the one reported by Jiang et al. for a spinal location, radiographs and CT did not allow characterization of the tissue nature of the lesion [4]. CT arthrography nonetheless delineated its multicompartmental extent and, through opacification of the subtalar joint and flexor hallucis longus tendon sheath following talocrural injection, demonstrated communication between these synovial compartments, which may have facilitated the extension of the lesion. On MRI, localized TGCT is generally seen as a discrete, well-circumscribed lobulated lesion abutting or wrapping around the involved tendon, whereas the diffuse form appears as infiltrative, poorly circumscribed synovial thickening that may extend across several synovial compartments, as observed in our patient with involvement of the flexor hallucis longus tendon sheath and the talocrural and subtalar joints. In both forms, signal is characteristically low across both T1- and T2-weighted sequences [7]. This low signal is explained by the paramagnetic behavior of iron within hemosiderin, which distorts the local magnetic environment and accelerates signal decay, an effect that becomes especially conspicuous as blooming on gradient-echo T2* sequences [8,9]. In our case, this combination of T1/T2 hypointensity and pronounced susceptibility artifact on gradient-echo imaging was clearly present and strongly suggestive of the diagnosis, a finding consistently reported across the literature, including in the recurrence case described by Chiang et al., where similar T1 hypointensity had likewise guided the diagnosis [6]. This blooming artifact correlated directly with the hemosiderin-laden macrophages identified on histological examination. Moderate post-contrast enhancement, as observed in our patient, is also a common though variable feature of these lesions [7]. Although highly suggestive, this imaging pattern does not obviate the need for histopathological confirmation, which remains the only means of establishing the diagnosis with certainty, as was the case here following surgical resection.

The main differential diagnoses and their distinguishing features are summarized in Table 1. Tendon sheath fibroma represents the main differential diagnosis for a tendon sheath lesion, as its dense collagen content may also produce low T1 and T2 signal; however, it lacks hemosiderin and therefore does not show blooming on gradient-echo sequences, and it typically presents as a small, well-circumscribed nodule with mild or absent enhancement, rather than the infiltrative multicompartmental involvement observed in our patient [7]. Synovial hemangioma and hemophilic arthropathy can also mimic TGCT on MRI, as they produce hemosiderin-related signal changes from repeated bleeding into the joint. However, synovial hemangioma typically shows high T2 signal with serpiginous vascular channels, phleboliths, and avid enhancement, whereas hemophilic arthropathy occurs in the context of a known coagulopathy and usually involves multiple joints, with cartilage loss and subchondral cysts [10]. In our patient, the absence of a known coagulopathy, of high T2 signal, and of phleboliths argued against these diagnoses. Synovial chondromatosis, including its tenosynovial form, may also present as multilobulated synovial proliferation; however, it usually shows multiple calcified chondral bodies with ring-and-arc mineralization, optimally depicted on CT, and its nonmineralized cartilage displays high signal intensity on T2-weighted images with peripheral and septal enhancement [11]. In our patient, the absence of calcification on radiographs and CT arthrography, together with the low T2 signal, argued against this diagnosis. 

**Table 1:** Main differential diagnoses of tenosynovial giant cell tumor on MRI TGCT: tenosynovial giant cell tumor; GRE: gradient echo Reference: [7,10,11]

Diagnosis	Clinical context	T1 signal	T2 signal	Blooming on GRE	Enhancement	Key distinguishing features
TGCT	Young to middle-aged adults; usually monoarticular	Low to intermediate	Low	Marked (none if small and focal)	Moderate, variable	Infiltrative, poorly circumscribed synovial thickening, mono or multicompartmental; bone erosions possible
Tendon sheath fibroma	Adults; most commonly in the hand	Low	Low to intermediate	Absent	Mild or absent	Small, well-circumscribed nodule attached to the tendon; no hemosiderin
Synovial hemangioma	Children and young adults	Intermediate, isointense to muscle	High, lobulated	Possible (prior hemorrhage)	Avid	Serpiginous vascular channels; phleboliths
Hemophilic arthropathy	Known coagulopathy; recurrent hemarthroses	Low	Low	Marked	Variable	Often polyarticular; cartilage loss, subchondral cysts
Synovial chondromatosis	Adults, male predominance; monoarticular	Low to intermediate	Very high	Possible at sites of calcification	Peripheral and septal	Multiple calcified chondral bodies with ring-and-arc mineralization, optimally seen on CT

Management options range from surgery to radiotherapy and, more recently, systemic pharmacological agents, used alone or combined depending on disease extent [12-14]; among these, surgery is generally favored as the initial approach [12].

Complete resection is generally more achievable in localized disease, which is associated with a lower recurrence rate than the diffuse form, whereas diffuse intra-articular involvement is more difficult to eradicate surgically and may warrant adjunct therapies such as radiotherapy or, more recently, targeted agents including tyrosine kinase inhibitors and CSF1 receptor-blocking monoclonal antibodies [13,14]. In our patient, surgery was chosen as first-line treatment following multidisciplinary team discussion, given the symptomatic lesion and the feasibility of complete excision through combined anterior and posteromedial approaches. As the diffuse form carries a higher risk of recurrence than localized disease, long-term clinical and MRI surveillance is required, and systemic therapy with CSF1 receptor inhibitors would be considered in case of recurrence or unresectable disease [13,14].

The main limitation of this report is the short postoperative follow-up of two months, as the six-month MRI had not yet been performed; recurrence therefore cannot be excluded at this stage.

## Conclusions

This case highlights the importance of considering diffuse-type tenosynovial giant cell tumor among the differential diagnoses of a synovial mass of the ankle, even in a location as rare as the flexor hallucis longus tendon sheath. In our patient, the post-traumatic context initially misled the work-up, and MRI played a central role owing to its ability to demonstrate the characteristic hemosiderin deposits, particularly on gradient-echo sequences.

Although highly suggestive on imaging, definitive diagnosis remains histopathological. In this case, complete surgical excision with negative margins was achieved; however, given the higher recurrence risk of the diffuse form, long-term clinical and MRI surveillance remains essential. This case therefore illustrates the diagnostic value of MRI in an unusual anatomical presentation of this entity, particularly when a soft-tissue abnormality persists or progresses despite an apparently plausible traumatic explanation.
